# Tobacco and electronic cigarette cues for smoking and vaping: an online experimental study

**DOI:** 10.1186/s13104-020-4899-3

**Published:** 2020-01-15

**Authors:** Anna K. M. Blackwell, Katie De-loyde, Laura A. Brocklebank, Olivia M. Maynard, Theresa M. Marteau, Gareth J. Hollands, Paul C. Fletcher, Angela S. Attwood, Richard W. Morris, Marcus R. Munafò

**Affiliations:** 1 School of Psychological Science, University of Bristol, 12a Priory Road, Bristol, BS8 1TU UK; 2 Behaviour and Health Research Unit, University of Cambridge, Cambridge, CB2 0SR UK; 3 Department of Psychiatry, Cambridgeshire and Peterborough NHS Foundation Trust, University of Cambridge, Douglas House, Cambridge, CB2 8AH UK; 4 The Wellcome-MRC Institute of Metabolic Science-Metabolic Research Laboratories (IMS-MRL), University of Cambridge, Cambridge, CB2 0QQ UK; 5 Bristol Medical School: Population Health Sciences, Canynge Hall, 39 Whatley Road, Bristol, BS8 2PS UK; 6 MRC Integrative Epidemiology Unit (IEU), University of Bristol, Oakfield House Oakfield Grove, Bristol, BS8 2BN UK

**Keywords:** Smoking, Cigarette, Vaping, E-cigarette, Cue reactivity, Public health, Policy

## Abstract

**Objective:**

This study examined whether exposure to smoking and vaping cues the urge to smoke or vape. It extends previous studies on first-generation cigalikes (visually similar to cigarettes) and second-generation devices (visually similar to pens) by including third-generation tank system devices (larger bulky units). In an online experiment, participants were randomly assigned to view one of four videos, which included smoking, vaping (cigalike or tank system), or neutral cues. The primary outcome was urge to smoke. Secondary outcomes were urge to vape, desire to smoke and vape, and intention to quit or remain abstinent from smoking.

**Results:**

UK adults varying in smoking (current or former) and vaping (user or non-user) status (n = 1120) completed the study: 184 (16%) failed study attention checks meaning 936 were included in the final analysis. Urges to smoke were similar across cue groups. Urges to vape were higher following exposure to vaping compared to neutral cues. There was no clear evidence of an interaction between cue group and smoking or vaping status. The lack of cueing effects on smoking urges is inconsistent with previous research, raising questions about the ability to assess craving in online settings.

## Introduction

The impact of e-cigarette use (vaping) is debated within global public health communities; there is evidence that it is an effective smoking cessation aid [1, 2], but there are also concerns around potential risks, including renormalisation of smoking [3]. In the UK, approximately 6% of adults (age 16 +) vape [4]. Public Health England (PHE) support e-cigarette use for smoking cessation [5], which is recommended as part of the National Health Service (NHS) smoke-free advice [6]. E-cigarettes are not included in the Health Act 2006, which bans smoking in public places; however, organisations often include them alongside cigarettes within smoking policies. There is also variability across organisations regarding the availability of shared or separate spaces for smokers and vapers [7].

The impact of differences in available spaces on smokers and vapers is unknown. Exposure to smoking or vaping cues has the potential to increase smoking or vaping urges, which is particularly concerning for former- or non-users in public areas. In shared spaces, exposure to smoking cues could increase smoking urges, or reduce vaping urges, among former smokers and those trying to quit through vaping, undermining smoking abstinence. It is also possible that exposure to vaping in shared spaces could cue vaping urges in smokers, increasing the desire to reduce or quit smoking through vaping.

Previous research on the impact of vaping cues has focussed on cross-cueing effects. For example, passive exposure to first-generation cigalike e-cigarettes (visually similar to cigarettes) can increase desire to vape and smoke, whereas exposure to tobacco cigarettes may only increase desire to smoke [8]. Similarly, second-generation e-cigarettes (size and shape of a large pen) can cue smoking urges among young adult daily and non-daily smokers [9]. The impact of newer third-generation e-cigarettes is currently unknown.

We aimed to directly compare the impact of viewing different e-cigarette cues (including first-generation cigalikes and third-generation tank systems, which are larger, bulky devices), to tobacco smoking or neutral cues among people with different smoking and vaping backgrounds. We hypothesised that smoking urges following exposure to vaping cues would be higher relative to neutral cues and lower relative to smoking cues, and that smoking urges would be lower following exposure to tank system vape cues relative to cigalike cues.

## Main text

### Methods

#### Study design

This online study used a between–subjects design. Participants were allocated to one of four stimulus groups (cigalike, tank system, tobacco cigarette, neutral) according to their smoking and vaping status (i. dual user: current smoker and vaper, ii. smoker: current smoker, non-vaper, iii. vaper: current vaper, former smoker, iv. non-user, former smoker, non-vaper). Participants were randomised to view one of four video cues [10–13]. Each video showed two individuals talking with one shown: i. vaping using a cigalike; ii. vaping using a tank system, iii. smoking a tobacco cigarette, or iv. moving their hand to their mouth without smoking or vaping (neutral). The primary outcome measure was post-intervention self-reported craving for tobacco cigarettes (smoking urge). Secondary outcomes were post-intervention self-reported craving for e-cigarettes (vaping urge), post-intervention desire for a cigarette and e-cigarette, intention to quit tobacco smoking (current smokers), and self-efficacy to remain abstinent from smoking (former smokers).

#### Participants

The study was hosted on the Qualtrics online survey platform [14]. Participants were UK adults (18 + years) who were either: current smokers (smoke ≥ 5 cigarettes a day for at least one year, not trying to quit) or former smokers (previously smoked ≥ 5 cigarettes a day for at least one year) and were either vapers (vape daily), or non-vapers (vaped ≤ 20 times). They were recruited through the Prolific crowdsourcing platform [15], which advertised the study to its members based on pre-specified screening questions. The study took approximately 10 min and participants were reimbursed £1 through their account.

#### Measures

Self-reported craving for tobacco was measured pre- and post-intervention using the brief questionnaire of smoking urges (QSU-Brief) [16, 17], with a modified version used to assess self-reported craving for e-cigarettes [18], on a scale of 10 (strongly disagree) to 70 (strongly agree). Desire for a cigarette and e-cigarette were collected pre- and post-intervention on a visual analogue scale (VAS) of 0–100. The following questions assessed intention to quit smoking or self-efficacy to remain abstinent: ‘Are you planning to quit smoking within the next 6 months?’ [19], ‘How confident are you that you will remain a non-smoker?’ [20], using scales of 1 (low) to 5 (high).

#### Procedure

Participants who expressed an interest were shown an information statement explaining the study. They were told the purpose was to provide feedback on videos for future research, to avoid them paying undue attention to their desire to smoke or vape. Participants willing to continue completed a tick box consent page. Participants then completed screening and demographic questions, baseline ratings of smoking and vaping craving, alongside filler questions. They then viewed one of four one-min cue videos according to their condition, followed by video-related cover questions and questions on craving and intention to quit smoking or remain abstinent. Finally, participants were asked what they thought the purpose was of the study. Attention check questions were embedded within the questions and participants who failed these were excluded post-randomisation and replaced to ensure high data quality. At the end of the study, participants were provided with debriefing information and contact details of the research team.

#### Data analysis

Two-way ANCOVAs were used to assess the impact of exposure to video cues (cigalike, tank system, cigarette, neutral) on: i. post-intervention smoking and vaping urge scores, and ii. post-intervention desire for a cigarette and e-cigarette scores, for the four smoking and vaping status groups (dual-user, smoker, vaper, non-user), using pre-intervention score as a covariate. Two-way ANOVAs were used to assess the impact of video cue on secondary outcomes (intention to quit or remain abstinent) for the four smoking and vaping status groups.

Further details of the study methods, materials, additional measures and statistical analysis plan can be found in the preregistered study protocol [21].

## Results

A total of 1120 participants completed the study. 184 (16%) failed attention checks and were excluded post-randomisation,^1^ meaning 936 participants were included in the final analysis (see Table 1 for demographic characteristics).

**Table 1:** Demographic, smoking and vaping characteristics of experimental groups (n = 936)

	Experimental groups n (%)^a^			
	Cigalike (n = 226)	Tank system (n = 232)	Cigarette (n = 242)	Neutral (n = 236)
Age (M, SD, range)	40 (12) 19–75	39 (13) 19–72	38 (11) 19–70	38 (10) 20–67
Gender				
Male	71 (31)	90 (39)	86 (36)	81 (34)
Female	155 (69)	142 (61)	156 (64)	155 (66)
Education				
Higher education or professional	102 (45)	112 (48)	117 (48)	116 (49)
A levels or equivalent	66 (29)	64 (28)	66 (27)	61 (26)
GCSE/O level grade A*–C, or equivalent	47 (21)	52 (22)	47 (19)	43 (18)
Qualifications at level 1 and below	3 (1)	1 (< 1)	3 (1)	7 (3)
Other qualifications: level unknown	7 (3)	0	4 (2)	4 (2)
No qualifications	1 (< 1)	3 (1)	5 (2)	5 (2)
Smoking and vaping status				
Dual users	23 (10)	28 (12)	20 (8)	25 (11)
Smokers only	67 (30)	68 (29)	71 (29)	72 (31)
Vapers only	66 (29)	68 (29)	76 (31)	72 (31)
Non-users	70 (31)	68 (29)	75 (31)	67 (28)

^a^Unless otherwise stated

### Smoking and vaping urges

There was no clear evidence of an interaction effect between video cue group and smoking and vaping status group for either ANCOVA models: smoking urge (F[9919] = 1.63, p = 0.10) and vaping urge (F[9919] = 0.66, p = 0.75). Therefore, the interaction was dropped from the model in favour of a single model to estimate main effects with greater precision.

There was no clear evidence of a main effect between the four video cue groups on smoking urge (F[3, 928] = 1.33, p = 0.26): there was no evidence of a difference in smoking urge between: i. vaping cues (combined) and neutral cue (mean difference [MD] = 0.05, 95% CI 0.89–0.98, p = 0.92), ii. vaping cues (combined) and smoking cue (MD = 0.9, 95% CI 0.03–1.83, p = 0.06), or iii. cigalike and tank system vaping cues (MD = 0.1, 95% CI 0.99–1.20, p = 0.85) (Table 2).

**Table 2:** Primary and secondary outcomes– adjusted

Cue type	Experimental groups M (95% CIs)			
	Cigalike	Tank system	Cigarette	Neutral
Urge to smoke^a^	25.7 (24.9, 26.5)	25.8 (25.0, 26.6)	26.6 (25.9, 27.4)	25.8 (25.0, 26.6)
Urge to vape^a^	25.2 (24.4, 25.9)	25.5 (24.7, 26.2)	24.1 (23.4, 24.9)	23.4 (22.6, 24.1)
Desire for a cigarette^b^	28.7 (26.6, 30.7)	28.9 (26.9, 30.9)	31.2 (29.2, 33.3)	28.7 (26.6, 30.7)
Desire for an e-cigarette^b^	26.6 (24.8, 28.4)	26.3 (24.6, 28.1)	25.1 (23.3, 26.8)	23.6 (21.9, 25.4)
Intention to quit smoking^c^	2.7 (2.4, 2.9)	2.9 (2.6, 3.1)	2.6 (2.3, 2.8)	2.7 (2.5, 3.0)
Likelihood remain abstinent^d^	4.3 (4.2, 4.5)	4.2 (4.1, 4.4)	4.4 (4.3, 4.6)	4.1 (3.9, 4.2)

^a^QSU (Questionnaire of smoking or vaping urges): 10 (minimum)–70 (maximum)

^b^Desire for a cigarette/e-cigarette: 0 (not at all)–100 (most ever)

^c^Intentions to quit smoking (current smokers): 1 (least)–5 (most)

^d^Likelihood to remain abstinent from smoking (ex-smokers): 1 (least)–5 (most)

There was strong evidence of a main effect between the video cue groups on vaping urge (F[3928] = 6.66, p < 0.001): urges were higher following exposure to both cigalike (MD = 1.8, 95% CI 0.4–3.2, p = 0.005) and tank system vaping (MD = 2.1, 95% CI 0.7–3.5, p < 0.001) cues compared to the neutral cue (Table 2).

### Desire to smoke and vape

There was no clear evidence of a main effect between the four video cue groups on desire to smoke (F[3928] = 1.58, p = 0.19) and weak evidence of a difference on desire to vape (F[3928] = 2.43, p = 0.06), which was higher following exposure to cigalike (MD = 3.0, 95% CI 0.6–5.4, p = 0.02) and tank system (MD = 2.7, 95% CI 0.3–5.1, p = 0.03) cues compared to the neutral cue (Table 2).

### Intention to quit or remain abstinent

There was no evidence of a main effect of video cue groups on intention to quit smoking (F[3369] = 1.33, p = 0.27). There was weak evidence of a main effect of video groups on intention to remain abstinent from smoking (F[3557] = 3.034, p = 0.03), which was higher following exposure to the cigarette cue compared to the neutral cue (MD = 0.3, 95% CI 0.03–0.6, p = 0.02) (Table 2).

The results remained unchanged when sensitivity analyses were conducted to remove participants who did not correctly identify the video cue (n = 88), or correctly identified the purpose of the study (n = 421).

The smoking and vaping urge and desire scores were heavily skewed towards the minimum scores (being 10 and 0 respectively), demonstrating possible floor effects. A comparison amongst video cue groups between those participants at floor or above found that the proportions were similar across groups.

## Discussion

This online study found no evidence of a cross-cueing effect of exposure to vaping cues on smoking urges or exposure to smoking cues on vaping urges, or an interaction between cue exposure effects and smoking or vaping status. Similarly, there was no evidence of a cueing effect on participants’ reported desire to smoke, or intention to quit smoking. There was some evidence of exposure to smoking, relative to neutral, cues increasing intention to remain abstinent in former smokers, and of vaping, relative to neutral, cues increasing vaping urges and desires.

The limited cueing effects on smoking urges were unexpected. One possibility is that previous studies have overestimated the impact on smoking urges of exposure to vaping or smoking cues; however, given differences in study methodology, it is more likely that the lack of effects reflect—at least in part—limitations of the materials and setting used in the current design. For example, King and colleagues [9] used confederate smokers or vapers, exposing participants to more ecologically valid cues compared to the videos used here.

Videos have been used in previous cue reactivity studies [22], and have the advantage of portraying contextual cues involved in typical, dynamically—represented smoking behaviour, although they only require participants to be passive observers [23]. In the present study the videos were specifically designed to show relatively neutral contexts, changing only the use of a cigarette, e-cigarette or neutral action, to control for confounding smoking cues (e.g., alcohol-related bar setting), which may have reduced their potential to elicit cravings. However, this study did find increased vaping urges following exposure to vaping video cues relative to neutral cues, which suggests the videos alone may not explain the lack of cueing effects on smoking urges.

It is possible that the online setting precluded control over other aspects of the environment in which participants completed the study, which could have influenced their responses. Both nicotine dependence and deprivation influence cue reactivity [24]. It is possible that these were both low among participants in the present study and that this reduced the likelihood of the videos cueing smoking and vaping urges. This explanation is consistent with the tendency towards floor effects found on the smoking and vaping scales of craving and desire. We did not measure nicotine dependence, and it was not possible to implement a minimum period of smoking or vaping abstinence before starting the study in the online setting; therefore, it is not possible to explore this further.

## Conclusions

The current study found no evidence that exposure to videos of smoking or vaping cued smoking urges and no evidence of interaction effects between cue exposure and smoking and vaping status. These findings may be explained as a consequence of using video cues with neutral contexts within an online setting, which allowed for minimal control over participants’ recent smoking or vaping activity. Future studies should exercise caution when using an artificial setting to assess craving.

## Limitations

- Using videos of smoking or vaping behaviour with limited contextual details may have been insufficient to elicit smoking cravings.
- It was not possible to control for a minimum period of smoking or vaping abstinence in the online setting.
- We did not measure nicotine dependence which could have influenced cue reactivity.

## Data Availability

The anonymised data collected are available as open data via the University of Bristol online data repository [10.5523/bris.299889i8ysm0d21dt3rz63nipg].

## Abbreviations

e-cigarette: electronic cigarette
UK: United Kingdom
PHE: Public Health England
NHS: National Health Service
ANCOVA: Analysis of Covariance
ANOVA: Analysis of Variance
QSU: questionnaire of smoking urges
VAS: visual analogue scale
M: mean
SD: standard deviation
A level: General Certificate of Education Advanced Level
O level: General Certificate of Education Ordinary Level
GCSE: General Certificate of Secondary Education
MD: mean difference
CI: confidence interval

## Footnotes

1 A higher number of non-vapers (smokers = 79, non-users = 73) were excluded compared to vapers (vapers = 13, dual-users = 19). This difference may at least partly reflect the placement of the second attention question within a series of vaping-related questions. Future studies should embed attention questions within neutral questions in which levels of attention would be expected to be similar across groups.
